# 3-(2-Ethyl-2-phenyl­hydrazin-1-yl­idene)indolin-2-one

**DOI:** 10.1107/S1600536812047988

**Published:** 2012-11-28

**Authors:** Uzma Ashiq, Rifat Ara Jamal, Hina Ismail, Khalid Mohammed Khan, Sammer Yousuf

**Affiliations:** a Department of Chemistry, University of Karachi, Karachi 75270, Pakistan; b H.E.J. Research Institute of Chemistry, International Center for Chemical and Biological Sciences, University of Karachi, Karachi 75270, Pakistan

## Abstract

In the title compound, C_16_H_15_N_3_O, the dihedral angle between the indole ring system (r.m.s. deviation = 0.020 Å) and the phenyl ring is 14.49 (9)°. The mol­ecular conformation is supported by an intra­molecular C—H⋯O inter­action, which closes an *S*(7) ring. In the crystal, inversion dimers linked by pairs of N—H⋯O hydrogen bonds generate *R*_2_^2^(8) loops.

## Related literature

For a related structure, see: Jamal *et al.* (2011 ▶). For background to Schiff bases, see: Chaluvaraju & Zaranappa (2011 ▶); Khan *et al.* (2009 ▶).
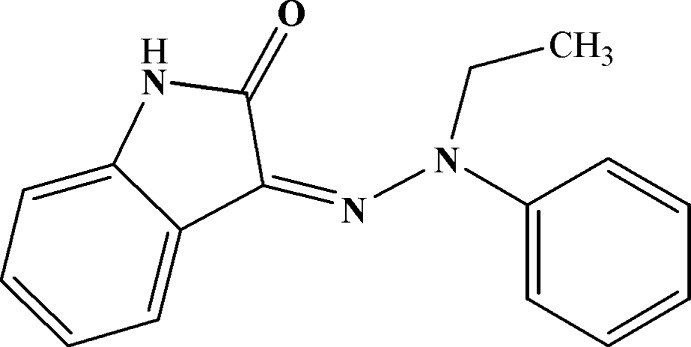


## Experimental

### 

#### Crystal data

C_16_H_15_N_3_O
*M* *_r_* = 265.31
Monoclinic, 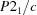

*a* = 9.463 (2) Å
*b* = 17.303 (4) Å
*c* = 8.5403 (18) Å
β = 104.427 (5)°
*V* = 1354.3 (5) Å^3^
*Z* = 4
Mo *K*α radiation
μ = 0.08 mm^−1^
*T* = 273 K
0.35 × 0.18 × 0.06 mm

#### Data collection

Bruker SMART APEX CCD diffractometer
Absorption correction: multi-scan (*SADABS*; Bruker, 2000 ▶) *T*_min_ = 0.971, *T*_max_ = 0.995
7875 measured reflections
2448 independent reflections
1783 reflections with *I* > 2σ(*I*)
*R*_int_ = 0.032

#### Refinement

*R*[*F* ^2^ > 2σ(*F* ^2^)] = 0.047
*wR*(*F* ^2^) = 0.116
*S* = 1.08
2448 reflections
181 parameters
H-atom parameters constrained
Δρ_max_ = 0.18 e Å^−3^
Δρ_min_ = −0.17 e Å^−3^

### 

Data collection: *SMART* (Bruker, 2000 ▶); cell refinement: *SAINT* (Bruker, 2000 ▶); data reduction: *SAINT*; program(s) used to solve structure: *SHELXS97* (Sheldrick, 2008 ▶); program(s) used to refine structure: *SHELXL97* (Sheldrick, 2008 ▶); molecular graphics: *SHELXTL* (Sheldrick, 2008 ▶); software used to prepare material for publication: *SHELXL97*.

## Supplementary Material

Crystal structure: contains datablock(s) global, I. DOI: 10.1107/S1600536812047988/hb6996sup1.cif

Structure factors: contains datablock(s) I. DOI: 10.1107/S1600536812047988/hb6996Isup2.hkl

Supplementary material file. DOI: 10.1107/S1600536812047988/hb6996Isup3.cml

Additional supplementary materials:  crystallographic information; 3D view; checkCIF report

## Figures and Tables

**Table 1:** Hydrogen-bond geometry (Å, °)

*D*—H⋯*A*	*D*—H	H⋯*A*	*D*⋯*A*	*D*—H⋯*A*
C15—H15*A*⋯O1	0.97	2.21	2.916 (2)	128
N1—H1*A*⋯O1^i^	0.86	1.99	2.844 (2)	172

Symmetry code: (i) 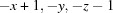.

**Fig. 1.**
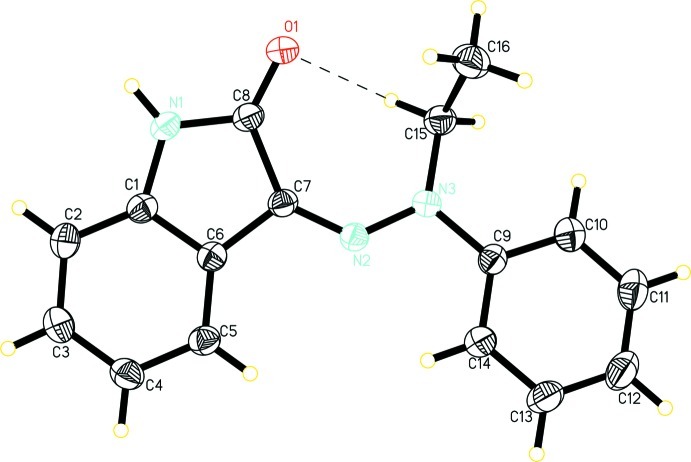
The molecular structure of (I) with displacement ellipsoids drawn at 30% probability level.

**Fig. 2.**
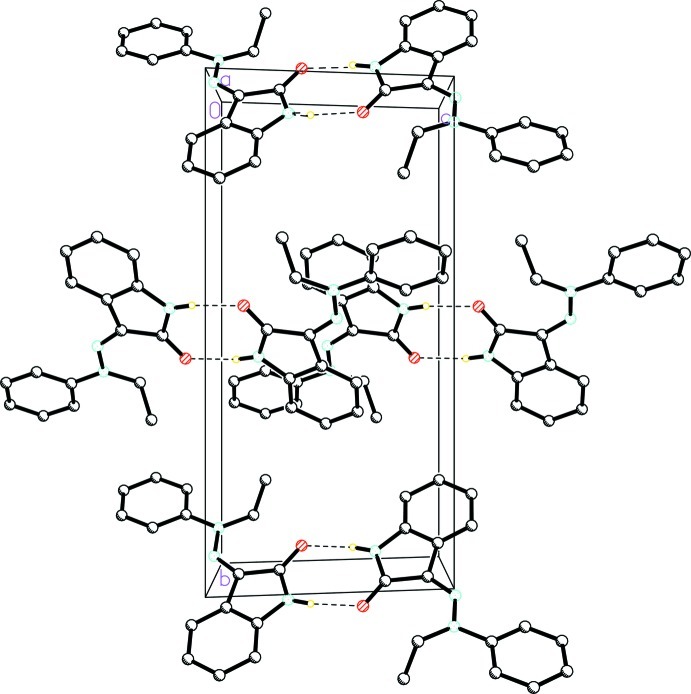
The crystal packing of the title compound. Hydrogen atoms are omitted for clearity.

## supplementary crystallographic information

### Comment

Isatin and its Schiff bases form an important class of organic compounds with a variety of biological activities. Many studies have reported regarding the biological activities of Schiff bases, including their antifungal, antibacterial, anticancer and antiglycation (Khan *et al.*, 2009; Chaluvaraju & Zaranappa. 2011). In order to study the biological activity of title compound, we undertook the synthesis of title compound and report its crystal structure in this paper (Fig. 1). The title compound I was found a potent DPPH radical scavenger.

The title compound, C_16_H_15_N_3_O is an structural analogue of our previously published compound 3-amino-*N*'-(2-oxoindolin-3-ylidene)- benzohydrazide (Jamal *et al.*, 2011) with the difference that the keto amine phenyl moiety is replaced by phenyl ring (C9–C14) and N3 is substituted with ethyl group (C15–C16). The phenyl and indole rings are each planar with the dihedral angle of 14.49 (9)° between them. The geometry of molecule is stabilized by an intramolecular C15—H15A···O1 hydrogen bond. In the crystal molecules are consolidated by intermolecular N1—H1A···O1 hydrogen bond (Fig. 2. symmetry codes as in Table 2).

### Experimental

To a solution of 2,3-Indolinedione (10 mmol, 1.47 g) in 15 ml of ethanol with few drops of glacial acetic acid and 1-ethyl-1-phenylhydrazine (10 mmol,1.36 g) in 15 ml e thanol were added. The mixture was refluxed for 24 h and a solid was obtained upon removal of the solvent by rotary evaporation.The resulting solid was washed with hexane to afford the title compound. Yellow plates were grown from a mixture of ethanol and methanol (1:1) solvents by slow evaporation at room temperature.

### Refinement

H atoms on methyl, methylene, phenyl and nitrogen were positioned geometrically with C—H = 0.96, 0.97, 0.93 and C—H = 0.86 Å respectively, and constrained to ride on their parent atoms with *U*_iso_(H)= 1.2*U*_eq_(CH, CH_2_ and NH) and 1.5*U*_eq_(CH_3_).

### Crystal data

**Table:**

C_16_H_15_N_3_O	*F*(000) = 560
*M**_r_* = 265.31	*D*_x_ = 1.301 Mg m^−^^3^
Monoclinic, *P*2_1_/*c*	Mo *K*α radiation, λ = 0.71073 Å
*a* = 9.463 (2) Å	Cell parameters from 1614 reflections
*b* = 17.303 (4) Å	θ = 2.7–28.2°
*c* = 8.5403 (18) Å	µ = 0.08 mm^−^^1^
β = 104.427 (5)°	*T* = 273 K
*V* = 1354.3 (5) Å^3^	Plate, yellow
*Z* = 4	0.35 × 0.18 × 0.06 mm

### Data collection

**Table:**

Bruker SMART APEX CCD diffractometer	2448 independent reflections
Radiation source: fine-focus sealed tube	1783 reflections with *I* > 2σ(*I*)
Graphite monochromator	*R*_int_ = 0.032
ω scan	θ_max_ = 25.5°, θ_min_ = 2.2°
Absorption correction: multi-scan (*SADABS*; Bruker, 2000)	*h* = −11→11
*T*_min_ = 0.971, *T*_max_ = 0.995	*k* = −20→20
7875 measured reflections	*l* = −10→10

### Refinement

**Table:**

Refinement on *F*^2^	Primary atom site location: structure-invariant direct methods
Least-squares matrix: full	Secondary atom site location: difference Fourier map
*R*[*F*^2^ > 2σ(*F*^2^)] = 0.047	Hydrogen site location: inferred from neighbouring sites
*wR*(*F*^2^) = 0.116	H-atom parameters constrained
*S* = 1.08	*w* = 1/[σ^2^(*F*_o_^2^) + (0.0444*P*)^2^ + 0.2688*P*] where *P* = (*F*_o_^2^ + 2*F*_c_^2^)/3
2448 reflections	(Δ/σ)_max_ < 0.001
181 parameters	Δρ_max_ = 0.18 e Å^−^^3^
0 restraints	Δρ_min_ = −0.17 e Å^−^^3^

### Special details

**Table:**

Geometry. All e.s.d.'s (except the e.s.d. in the dihedral angle between two l.s. planes) are estimated using the full covariance matrix. The cell e.s.d.'s are taken into account individually in the estimation of e.s.d.'s in distances, angles and torsion angles; correlations between e.s.d.'s in cell parameters are only used when they are defined by crystal symmetry. An approximate (isotropic) treatment of cell e.s.d.'s is used for estimating e.s.d.'s involving l.s. planes.
Refinement. Refinement of *F*^2^ against ALL reflections. The weighted *R*-factor *wR* and goodness of fit *S* are based on *F*^2^, conventional *R*-factors *R* are based on *F*, with *F* set to zero for negative *F*^2^. The threshold expression of *F*^2^ > σ(*F*^2^) is used only for calculating *R*-factors(gt) *etc*. and is not relevant to the choice of reflections for refinement. *R*-factors based on *F*^2^ are statistically about twice as large as those based on *F*, and *R*- factors based on ALL data will be even larger.

### Fractional atomic coordinates and isotropic or equivalent isotropic displacement parameters (Å^2^)

**Table:**

	*x*	*y*	*z*	*U*_iso_*/*U*_eq_	
O1	0.65674 (17)	0.05361 (9)	−0.36767 (16)	0.0663 (5)	
N1	0.52529 (18)	−0.04875 (9)	−0.31253 (18)	0.0506 (5)	
H1A	0.4712	−0.0548	−0.4088	0.061*	
N2	0.79812 (16)	0.02839 (8)	0.01956 (17)	0.0389 (4)	
N3	0.89404 (17)	0.08210 (9)	0.00675 (17)	0.0426 (4)	
C1	0.5134 (2)	−0.09387 (10)	−0.1807 (2)	0.0419 (5)	
C2	0.4213 (2)	−0.15483 (11)	−0.1754 (2)	0.0508 (5)	
H2B	0.3551	−0.1723	−0.2682	0.061*	
C3	0.4310 (2)	−0.18901 (12)	−0.0267 (3)	0.0547 (6)	
H3A	0.3707	−0.2306	−0.0194	0.066*	
C4	0.5286 (2)	−0.16256 (11)	0.1112 (2)	0.0515 (5)	
H4A	0.5317	−0.1859	0.2101	0.062*	
C5	0.6221 (2)	−0.10171 (11)	0.1042 (2)	0.0446 (5)	
H5A	0.6884	−0.0844	0.1971	0.053*	
C6	0.6148 (2)	−0.06736 (10)	−0.0437 (2)	0.0392 (4)	
C7	0.6978 (2)	−0.00462 (10)	−0.0925 (2)	0.0397 (5)	
C8	0.6314 (2)	0.00580 (11)	−0.2721 (2)	0.0473 (5)	
C9	0.9830 (2)	0.10795 (10)	0.1577 (2)	0.0408 (5)	
C10	1.0982 (2)	0.15798 (12)	0.1632 (2)	0.0526 (5)	
H10A	1.1160	0.1764	0.0676	0.063*	
C11	1.1871 (2)	0.18077 (13)	0.3106 (3)	0.0639 (6)	
H11A	1.2647	0.2141	0.3128	0.077*	
C12	1.1626 (3)	0.15511 (13)	0.4522 (3)	0.0685 (7)	
H12A	1.2232	0.1703	0.5507	0.082*	
C13	1.0470 (3)	0.10645 (13)	0.4474 (3)	0.0661 (7)	
H13A	1.0295	0.0888	0.5437	0.079*	
C14	0.9564 (2)	0.08325 (11)	0.3022 (2)	0.0524 (5)	
H14A	0.8775	0.0510	0.3012	0.063*	
C15	0.9259 (2)	0.10639 (11)	−0.1461 (2)	0.0485 (5)	
H15A	0.8863	0.0684	−0.2289	0.058*	
H15B	1.0308	0.1079	−0.1319	0.058*	
C16	0.8632 (2)	0.18441 (12)	−0.2025 (3)	0.0636 (6)	
H16A	0.8869	0.1974	−0.3022	0.095*	
H16B	0.9037	0.2226	−0.1222	0.095*	
H16C	0.7592	0.1831	−0.2190	0.095*	

### Atomic displacement parameters (Å^2^)

**Table:**

	*U*^11^	*U*^22^	*U*^33^	*U*^12^	*U*^13^	*U*^23^
O1	0.0818 (11)	0.0690 (10)	0.0388 (8)	−0.0259 (9)	−0.0025 (7)	0.0131 (7)
N1	0.0587 (11)	0.0518 (10)	0.0347 (8)	−0.0096 (9)	−0.0008 (7)	−0.0005 (7)
N2	0.0412 (9)	0.0364 (8)	0.0376 (8)	0.0002 (7)	0.0070 (7)	0.0019 (7)
N3	0.0475 (10)	0.0431 (9)	0.0362 (8)	−0.0058 (8)	0.0084 (7)	0.0035 (7)
C1	0.0452 (11)	0.0397 (10)	0.0392 (10)	0.0032 (9)	0.0075 (8)	−0.0009 (8)
C2	0.0502 (13)	0.0467 (12)	0.0536 (12)	−0.0066 (10)	0.0091 (10)	−0.0082 (10)
C3	0.0551 (13)	0.0460 (12)	0.0640 (14)	−0.0083 (10)	0.0167 (11)	0.0001 (10)
C4	0.0528 (13)	0.0500 (12)	0.0532 (12)	0.0005 (10)	0.0158 (10)	0.0110 (10)
C5	0.0427 (11)	0.0486 (11)	0.0405 (11)	0.0037 (9)	0.0068 (8)	0.0036 (9)
C6	0.0381 (10)	0.0375 (10)	0.0406 (10)	0.0039 (8)	0.0073 (8)	0.0005 (8)
C7	0.0434 (11)	0.0384 (10)	0.0354 (9)	0.0015 (9)	0.0063 (8)	0.0015 (8)
C8	0.0552 (13)	0.0466 (11)	0.0362 (10)	−0.0032 (10)	0.0040 (9)	0.0017 (9)
C9	0.0418 (11)	0.0359 (10)	0.0422 (10)	0.0025 (9)	0.0058 (8)	−0.0015 (8)
C10	0.0495 (12)	0.0522 (12)	0.0559 (13)	−0.0055 (10)	0.0124 (10)	−0.0013 (10)
C11	0.0513 (14)	0.0584 (14)	0.0742 (16)	−0.0082 (11)	0.0010 (11)	−0.0089 (12)
C12	0.0771 (17)	0.0580 (14)	0.0557 (14)	−0.0023 (13)	−0.0113 (12)	−0.0086 (11)
C13	0.0910 (18)	0.0603 (14)	0.0404 (12)	−0.0040 (14)	0.0042 (11)	−0.0006 (10)
C14	0.0635 (14)	0.0495 (12)	0.0422 (11)	−0.0071 (11)	0.0095 (10)	−0.0004 (9)
C15	0.0481 (12)	0.0550 (12)	0.0426 (11)	−0.0001 (10)	0.0119 (9)	0.0024 (9)
C16	0.0624 (15)	0.0605 (14)	0.0656 (14)	0.0000 (12)	0.0113 (11)	0.0163 (11)

### Geometric parameters (Å, º)

**Table:**

O1—C8	1.227 (2)	C7—C8	1.517 (2)
N1—C8	1.359 (2)	C9—C10	1.384 (3)
N1—C1	1.398 (2)	C9—C14	1.387 (3)
N1—H1A	0.8600	C10—C11	1.386 (3)
N2—C7	1.300 (2)	C10—H10A	0.9300
N2—N3	1.323 (2)	C11—C12	1.361 (3)
N3—C9	1.425 (2)	C11—H11A	0.9300
N3—C15	1.472 (2)	C12—C13	1.373 (3)
C1—C2	1.376 (3)	C12—H12A	0.9300
C1—C6	1.393 (2)	C13—C14	1.380 (3)
C2—C3	1.383 (3)	C13—H13A	0.9300
C2—H2B	0.9300	C14—H14A	0.9300
C3—C4	1.381 (3)	C15—C16	1.504 (3)
C3—H3A	0.9300	C15—H15A	0.9700
C4—C5	1.386 (3)	C15—H15B	0.9700
C4—H4A	0.9300	C16—H16A	0.9600
C5—C6	1.382 (2)	C16—H16B	0.9600
C5—H5A	0.9300	C16—H16C	0.9600
C6—C7	1.460 (3)		
			
C8—N1—C1	112.74 (15)	C10—C9—C14	118.60 (18)
C8—N1—H1A	123.6	C10—C9—N3	120.67 (17)
C1—N1—H1A	123.6	C14—C9—N3	120.73 (17)
C7—N2—N3	129.64 (15)	C9—C10—C11	120.3 (2)
N2—N3—C9	114.13 (14)	C9—C10—H10A	119.9
N2—N3—C15	124.76 (15)	C11—C10—H10A	119.9
C9—N3—C15	120.49 (16)	C12—C11—C10	121.0 (2)
C2—C1—C6	122.30 (17)	C12—C11—H11A	119.5
C2—C1—N1	129.31 (17)	C10—C11—H11A	119.5
C6—C1—N1	108.40 (16)	C11—C12—C13	119.0 (2)
C1—C2—C3	117.40 (18)	C11—C12—H12A	120.5
C1—C2—H2B	121.3	C13—C12—H12A	120.5
C3—C2—H2B	121.3	C12—C13—C14	121.1 (2)
C4—C3—C2	121.2 (2)	C12—C13—H13A	119.4
C4—C3—H3A	119.4	C14—C13—H13A	119.4
C2—C3—H3A	119.4	C13—C14—C9	120.0 (2)
C3—C4—C5	120.91 (19)	C13—C14—H14A	120.0
C3—C4—H4A	119.5	C9—C14—H14A	120.0
C5—C4—H4A	119.5	N3—C15—C16	112.86 (17)
C6—C5—C4	118.62 (18)	N3—C15—H15A	109.0
C6—C5—H5A	120.7	C16—C15—H15A	109.0
C4—C5—H5A	120.7	N3—C15—H15B	109.0
C5—C6—C1	119.53 (18)	C16—C15—H15B	109.0
C5—C6—C7	132.28 (16)	H15A—C15—H15B	107.8
C1—C6—C7	108.18 (15)	C15—C16—H16A	109.5
N2—C7—C6	117.53 (15)	C15—C16—H16B	109.5
N2—C7—C8	137.30 (17)	H16A—C16—H16B	109.5
C6—C7—C8	105.09 (15)	C15—C16—H16C	109.5
O1—C8—N1	123.66 (17)	H16A—C16—H16C	109.5
O1—C8—C7	130.73 (18)	H16B—C16—H16C	109.5
N1—C8—C7	105.54 (16)		
			
C7—N2—N3—C9	176.71 (17)	C1—N1—C8—O1	−177.0 (2)
C7—N2—N3—C15	−12.3 (3)	C1—N1—C8—C7	0.2 (2)
C8—N1—C1—C2	−178.8 (2)	N2—C7—C8—O1	−1.0 (4)
C8—N1—C1—C6	1.2 (2)	C6—C7—C8—O1	175.5 (2)
C6—C1—C2—C3	0.9 (3)	N2—C7—C8—N1	−178.0 (2)
N1—C1—C2—C3	−179.08 (19)	C6—C7—C8—N1	−1.4 (2)
C1—C2—C3—C4	0.5 (3)	N2—N3—C9—C10	173.23 (16)
C2—C3—C4—C5	−1.3 (3)	C15—N3—C9—C10	1.8 (3)
C3—C4—C5—C6	0.6 (3)	N2—N3—C9—C14	−6.5 (3)
C4—C5—C6—C1	0.7 (3)	C15—N3—C9—C14	−177.88 (17)
C4—C5—C6—C7	−178.50 (19)	C14—C9—C10—C11	1.9 (3)
C2—C1—C6—C5	−1.6 (3)	N3—C9—C10—C11	−177.84 (18)
N1—C1—C6—C5	178.46 (17)	C9—C10—C11—C12	−0.5 (3)
C2—C1—C6—C7	177.86 (17)	C10—C11—C12—C13	−0.5 (4)
N1—C1—C6—C7	−2.1 (2)	C11—C12—C13—C14	0.2 (4)
N3—N2—C7—C6	174.94 (17)	C12—C13—C14—C9	1.2 (3)
N3—N2—C7—C8	−8.8 (4)	C10—C9—C14—C13	−2.2 (3)
C5—C6—C7—N2	−1.2 (3)	N3—C9—C14—C13	177.49 (18)
C1—C6—C7—N2	179.53 (16)	N2—N3—C15—C16	106.2 (2)
C5—C6—C7—C8	−178.5 (2)	C9—N3—C15—C16	−83.3 (2)
C1—C6—C7—C8	2.2 (2)		

### Hydrogen-bond geometry (Å, º)

**Table:**

*D*—H···*A*	*D*—H	H···*A*	*D*···*A*	*D*—H···*A*
C15—H15*A*···O1	0.97	2.21	2.916 (2)	128
N1—H1*A*···O1^i^	0.86	1.99	2.844 (2)	172

Symmetry code: (i) −*x*+1, −*y*, −*z*−1.

## References

[bb1] Bruker (2000). *SADABS*, *SMART* and *SAINT* Bruker AXS Inc., Madison, Wisconsin, USA.

[bb2] Chaluvaraju, K. C. & Zaranappa (2011). *Res. J. Pharm. Biol. Chem. Sci.* **2**, 541–546.

[bb3] Jamal, R. A., Ashiq, U., Yousuf, S. & Ain, Q. ul (2011). *Acta Cryst* E**67**, o2166. 10.1107/S1600536811029242 PMC3213601 22091178

[bb4] Khan, K. M., Khan, M., Ali, M., Taha, M., Rasheed, S., Perveen, S. & Choudhary, M. I. (2009). *Bioorg. Med. Chem.* **17**, 7795–7801. 10.1016/j.bmc.2009.09.028 19837595

[bb5] Sheldrick, G. M. (2008). *Acta Cryst* A**64**, 112–122. 10.1107/S0108767307043930 18156677

